# Recurrent hospitalization and healthcare resource use among patients with deep vein thrombosis and pulmonary embolism: findings from a multi-payer analysis

**DOI:** 10.1007/s11239-014-1108-z

**Published:** 2014-07-31

**Authors:** Kathleen Lang, Aarti A. Patel, Michael Munsell, Brahim K. Bookhart, Samir H. Mody, Jeff R. Schein, Joseph Menzin

**Affiliations:** 1 Boston Health Economics, Inc, 20 Fox Road, Waltham, MA 02451 USA; 2 Janssen Scientific Affairs LLC, 1000 Route 202, Raritan, NJ 08869 USA

**Keywords:** Venous thromboembolism, Resource use, Recurrence, Antithrombotic, Economics

## Abstract

The objective of this study was to assess deep vein thrombosis and pulmonary embolism (DVT/PE) recurrence rates and resource utilization among patients with an initial DVT or PE event across multiple payer perspectives. Retrospective analyses were performed using a software tool that analyzes health plan claims to evaluate treatment patterns and resource utilization for various cardiovascular conditions. Six databases were analyzed from three payer perspectives (Commercial, Medicare, and Medicaid). Patients were ≥18 years old with a primary diagnosis of DVT or PE associated with an inpatient and/or emergency room claim, had received an antithrombotic within 7 days before or 14 days after index, and had no diagnosis of atrial fibrillation during follow-up. Outcomes were assessed over a 1 year period following index. More PE patients were hospitalized for their index event than DVT patients (42–59 % DVT and 69–86 % PE) and had longer mean length of stay (2.35–2.95 days DVT and 3.26–3.76 days PE). Recurrent event rates among PE patients (12–32 %) were higher than those for DVT patients (6–16 %) across all payers. The highest rate of recurrence was observed among the Medicaid population [23 % overall (VTE); 16 % DVT; 32 % PE]. All-cause hospitalization in the year following their VTE episode occurred in 23–67 % DVT patients and 30–68 % PE patients. Medicaid had the highest proportion of patients with hospitalizations and ER visits. Recurrent VTE events and all-cause hospitalizations are relatively common, especially for patients who had a PE, and among those in the Medicaid payer population.

## Introduction

Venous thromboembolism (VTE) is a medical condition referring to all thrombosis (i.e., blood clots) of the veins, consisting of both deep vein thrombosis (DVT) and pulmonary embolism (PE). DVT most commonly occurs in the deep veins of the leg, although it can also occur in veins of the upper extremities, the pelvis, abdomen, and cerebral venous sinuses. PE, a more severe manifestation of VTE, includes either the formation of a thrombosis in blood vessels or the embolization of thrombi from other areas of the body into pulmonary circulation. It is estimated that the annual incidence of DVT/PE is approximately 1–2 persons per 1,000 with the risk increasing significantly with age, presence of cancer, recent surgery, or prior DVT or PE events [1–3].

With more than half of DVT/PE cases being hospital-acquired [4], DVT/PE is associated with substantial healthcare resource utilization and costs. A retrospective analysis of administrative claims data from 2004–2008 estimated that patients with a DVT or PE event had approximately $16,000 higher mean annual all-cause medical costs compared to matched patients without DVT or PE ($33,000 vs. $17,000), with hospitalizations representing the primary driver of the difference in cost ($17,174 vs. $6,515) [5].

The recurrence rate for DVT/PE is relatively high, estimated at between 5 and 10 % within the first year after an initial DVT or PE event, with recurrence rates up to 30 % within 8 years [6–8]. A study by Bullano et al. found that 12.1 and 18.5 % of DVT and PE patients experienced a recurrent VTE hospitalization over a 21-months follow-up period, respectively. Patients with a recurrent event experienced an average of 1.26 hospitalizations during follow-up, with each hospitalization averaging $12,326 per event [9]. An analysis of hospital administrative claims data by Spyropolous [10] found that within 1 year of an initial DVT or PE event, 5.3 and 14.3 % of patients experienced a hospitalization with a primary or secondary diagnosis of DVT/PE, respectively, with the mean cost of readmission being higher than the index event.

DVT/PE events and associated readmissions represent a particular challenge to the provision of quality care for U.S. hospitals and healthcare providers, with many health policy organizations implementing quality metrics aimed at reducing the incidence of preventable rehospitalizations, including organizations such as the Joint Commission, National Quality Forum, and Centers for Medicare and Medicaid Services [11, 12]. Given the relatively high likelihood of recurrence among DVT/PE patients, and the recent focus on quality measures in the evaluation of healthcare, robust data on the healthcare resource use associated with DVT/PE and DVT/PE recurrence could prove useful to various health plans charged with evaluating quality metrics.

Recent published data on the risk of recurrent DVT or PE and associated resource use across multiple payer perspectives are limited. A 2012 publication by Lefebvre [5] used data from 2008 or earlier on commercial, Medicare, and Medicaid beneficiaries to assess the healthcare costs and risk of recurrence for patients diagnosed with DVT or PE; however, results were not stratified by payer type. Another 2012 study of VTE recurrence and healthcare utilization evaluated differences between Medicaid and commercially insured patients, but only included patients aged less than 18 years [13]. Given this data gap, the objective of this study was to assess DVT/PE recurrence rates and resource utilization, including all-cause 30 days readmissions rates, all-cause hospitalizations and all-cause ER visits, among patients with an initial DVT or PE event across multiple payer perspectives.

## Methods

### Overview

This retrospective database analysis used 2 years of administrative claims records from six different databases and three different payer perspectives: *Commercial perspective*—IMS LifeLink Database (IMS), Clinformatics™ DataMart, a product of OptumInsight Life Sciences, Inc. (Eden Praire, MN) (Optum), Truven MarketScan^®^ Commercial Database (MSCommercial); *Medicare perspective*—Truven MarketScan^®^ Medicare Supplemental Database (MSMedicare), the Humana Medicare database (Humana); *Medicaid* *perspective*—Medicaid database for a southern US state (Medicaid). Given the large sample sizes in the IMS, Optum, MSCommercial, MSMedicare, and Humana databases, a 10 % random sample was selected from each of these, while the full Medicaid sample was used.

Patients included in the analysis had a primary diagnosis of either DVT or PE associated with an inpatient and/or emergency room claim (index date), had received an antithrombotic agent within 7 days before or 14 days after index, and had no diagnosis of atrial fibrillation during a 1 year period after index (follow-up). Study measures included demographics (e.g., age and sex), index hospitalization length of stay (LOS), recurrent DVT/PE-related hospitalizations, all-cause 30 days readmission rates, and the frequency of all-cause hospitalizations and ER visits during follow-up.

A condition-specific software tool that analyzes health plan claims to evaluate treatment patterns and resource utilization for various cardiovascular conditions was used for this analysis [Anticoagulant quality improvement analyzer (AQuIA)]. The tool provides a common analysis platform, which ensures that various population health data are evaluated in a consistent way. All logic utilized by the tool for patient selection and measure creation are similar to that of traditional retrospective database analyses, and are documented in the *patient selection* and *study measures* sections of this paper. The goal of utilizing this tool was to eliminate variations in outcome definitions and methodology, and focus on understanding how findings vary across populations that differ based on age, comorbidities, and other factors.

### Data sources

This study used six different de-identified, integrated databases including medical and pharmacy claims. Diagnoses and procedures were identified based on International classification of diseases ninth revision clinical modification (ICD-9-CM) and current procedural terminology (CPT) codes from patients’ medical claims, while medication use was assessed based on national drug codes (NDC) from patients’ pharmacy claims.

The IMS LifeLink^®^ Health Plan Claims Database is a commercial database consisting of approximately 55 million patients from over 75 managed care organizations across the US and several million Medicare managed-care enrollees from four US geographical regions. The Clinformatics™ DataMart database, a product of OptumInsight Life Sciences, Inc. (Eden Prairie, MN), consists of a single, large insurance healthcare plan that includes more than 53 million unique members spanning over 12 years. The Truven MarketScan^®^ Commercial Claims and Encounter Database is constructed from privately insured paid medical and prescription drug claims for approximately 30 million employees and their dependents (in 2010) [14]. The MarketScan Medicare Supplemental Database contains the healthcare experience of individuals with Medicare supplemental insurance paid by employers for approximately 3.42 million retirees (in 2010) [14]. The Humana database consists of the entire fully-insured commercial and Medicare members belonging to the Humana health plan. The database includes 3.4 million Medicare members, including 1.4 million Medicare advantage prescription drug members; only data from Medicare members of the Humana health plan were used in this analysis. The Medicaid program database from a southeastern US state used in this analysis covers low-income or disabled individuals and consists of two files: a claims file, with details on medical and pharmacy utilization; as well as an eligibility file, with details on monthly enrollment and patient demographics.

Two-years of claims available from each database were used in this study; IMS, 07/2010–06/2012; Optum, 04/2010–03/2012; MSCommercial, 07/2009–06/2011; MSMedicare, 07/2009–06/2011; Humana, 07/2010–6/2012; and Medicaid, 07/2008–06/2010.

### Patient selection

Patients were included in the study if they were ≥18 years of age, had at least one primary diagnosis of VTE (i.e., DVT or PE) associated with an inpatient and/or emergency room visit, had received an antithrombotic agent within 7 days before or 14 days after index, and did not have a diagnosis for atrial fibrillation (ICD-9-CM code 427.31) during the follow-up period (i.e., 1 year post index). The index date was defined as the date of the first DVT/PE diagnosis observed in the available data, identified by the following ICD-9-CM codes (DVT–451.1x, 451.2x, 453.0x, 453.2, 453.3x, 453.40, 453.41, 453.42, 453.8x, 997.2x; PE–415.1x). Patients were classified into mutually exclusive cohorts (i.e., DVT or PE) based on the diagnosis code associated with the index claim. Receipt of antithrombotic agents were identified by NDC codes.

Patients were followed over a period of 1 year after the index date (i.e., the follow-up period). All study measures were evaluated during the follow-up period. Additional criteria for continuous eligibility were not applied; however, patients who were Medicare and/or health maintenance organization eligible anytime during the 2-years period were excluded in the Medicaid database, in order to ensure the availability of all claims within this database.

### Study measures

Demographics (i.e., age and sex) and comorbidities at time of index were evaluated for all patients meeting inclusion criteria. The LOS of the index DVT/PE hospitalization was calculated among patients experiencing an inpatient hospitalization as their first DVT/PE claim. LOS was calculated as the difference between the discharge and admission dates recorded on the index inpatient stay.

The percentage of patients experiencing a recurrent DVT/PE-related hospitalization during the follow-up period was also evaluated. Patients with a primary DVT/PE coded inpatient visit (determined by the same ICD-9-CM codes used for index event identification) at any point after the initial index event were flagged as experiencing a recurrent hospitalization. The percentage of patients with any (i.e., not disease-specific) inpatient hospitalization, inpatient hospitalization within 30 days of index, and ER visit were reported for the follow-up period, as well as the mean number of visits per patient (among those experiencing an event) for each resource category. All-cause inpatient hospitalizations and ER visits were identified based on inpatient hospital/ER visit CPT or UB-92 revenue codes.

### Data analyses

All analyses were descriptive in nature. Categorical variables were summarized using counts and sample proportions. Mean values were reported for continuous measures. Study measures were reported for the overall study cohort (i.e., overall VTE), as well as stratified by index event diagnosis (i.e., DVT or PE).

SAS software (Version 9.3, SAS Institute, Cary, NC) was used for extracting medical/pharmacy claims and demographic information from all databases, and for organizing the data in the proper format to be utilized by the AQuIA software tool, which carried out the analyses that produced the study outcomes. The software tool is condition-specific and enables uploading pharmacy and medical claims data via a simple point-and-click method to produce results for a series of predetermined measures and generate sample-specific reports. The programming logic behind all predetermined measures has been outlined in the sections above. All personal identifiers were removed before data were uploaded to the tool.

## Result

### Demographic and clinical characteristics

The number of patients meeting the cohort selection criteria varied across databases; 1,055 IMS (327 DVT; 728 PE), 505 Optum (160 DVT; 345 PE), 1,541 MSCommercial (548 DVT; 993 PE), 510 MSMedicare (198 DVT; 312 PE), 697 Humana (198 DVT; 499 PE), and 249 Medicaid (146 DVT; 103 PE). Average age varied between 47–77 years for patients with DVT, and between 46–75 years among patients diagnosed with PE, with Medicaid typically having a younger population, and the Medicare databases (MSMedicare and Humana) having an older cohort. On average, more than half of study patients were female (Table 1).

**Table 1:** Patient demographics and clinical characteristics

Characteristics	IMS		Optum		MSCommercial		MSMedicare		Humana		Medicaid	
	DVT	PE	DVT	PE	DVT	PE	DVT	PE	DVT	PE	DVT	PE
Number of patients	327	728	160	345	548	993	198	312	198	499	146	103
Mean age	57.84	55.78	50.14	50.93	49.11	48.74	77.07	74.8	74.42	73.12	47.36	46.14
Female (%)	63	60	53	49	57	50	58	53	59	55	58	71
Age Groups												
18–29(%)	3	4	8	5	6	7	0	0	0	0	13	14
30–39(%)	13	6	12	14	13	13	0	0	0	0	15	17
40–49(%)	9	26	28	25	26	26	0	0	0	0	25	26
50–59(%)	19	22	23	30	36	33	0	0	0	0	27	29
60–64(%)	22	18	21	12	19	18	1	1	5	5	16	11
65+	34	24	9	14	1	2	99	99	65	65	5	3
Comorbidities (%)												
COPD	3	6	3	5	3	4	10	13	14	19	13	27
Hypertension	59	60	43	48	41	43	66	61	80	83	73	72
Cancer	53	44	32	38	34	33	45	46	47	50	28	44
Diabetes	25	24	11	18	16	18	29	26	38	36	40	36
Heart failure	9	10	6	7	6	7	11	20	24	23	25	20
Previous AMI	0	1	1	2	1	2	3	3	6	8	4	2
Coronary artery disease	9	12	12	8	9	9	26	27	26	36	18	25
Hyperlipidemia	34	41	34	40	30	30	38	37	60	63	43	40
Chronic kidney disease	16	9	9	7	6	6	19	13	32	24	21	12
Valvular disease	0	26	10	20	9	14	14	16	21	21	18	30
Diabetic neuropathy	0	1	3	1	1	2	6	2	8	7	10	8
Peripheral vascular disease	3	7	9	5	9	4	20	14	26	19	21	12
Retinopathy	0	0	0	1	0	1	3	1	5	2	4	2

*Data source* IMS, 07/2010–06/2012; Optum, 04/2010–03/2012; MSCommercial, 07/2009–06/2011; MSMedicare, 07/2009–06/2011; Humana, 07/2010–06/2012; Medicaid, 07/2008–06/2010

*DVT* deep vein thrombosis, *PE* pulmonary embolism, *COPD* chronic obstructive pulmonary disease, *AMI* acute myocardial infarction

Averaging across all databases, more than 50 % of patients meeting inclusion criteria had hypertension (41–80 % DVT; 43–83 % PE), and over 40 % had cancer (32–53 % DVT; 33–50 % PE). Diabetes (11–40 % DVT; 18–36 % PE) and hyperlipidemia (30–60 % DVT; 30–63 % PE) were other commonly observed comorbid conditions.

### Index hospitalization and recurrent VTE-related hospitalizations

The majority of patients identified with an index PE event were associated with an inpatient hospitalization (69–86 % across all databases) as opposed to during an ER visit only (14–31 %), while patients identified with an index DVT event had an equal likelihood of receiving an index diagnosis in either place of service (48 % index event in inpatient hospitalization across commercial plans (weighted average); 57 % across Medicare databases; 42 % Medicaid). Patients with an index hospitalization for PE had longer lengths of stay for the index event than those with DVT (2.35–2.95 days for DVT vs. 3.26–3.76 days for PE), with relatively consistent findings across all databases (Table 2).

**Table 2:** Average length of stay among patients with DVT or PE index hospitalization

Measure	IMS		Optum		MSCommercial		MSMedicare		Humana		Medicaid	
	DVT	PE	DVT	PE	DVT	PE	DVT	PE	DVT	PE	DVT	PE
Number of patients	327	728	160	345	548	993	198	312	198	499	146	103
Has index hospitalization												
N	163	621	74	266	261	763	117	241	107	427	61	71
%	50	85	46	77	48	77	59	77	54	86	42	69
Mean length of stay	2.35	3.26	2.65	3.76	2.59	3.46	2.91	3.57	2.84	3.29	2.95	3.64

*Data source* IMS, 07/2010-06/2012; Optum, 04/2010–03/2012; MSCommercial, 07/2009–06/2011; MSMedicare, 07/2009–06/2011; Humana, 07/2010–06/2012; Medicaid, 07/2008–06/2010

*DV* deep vein thrombosis, *PE* pulmonary embolism

**Fig. 1:**
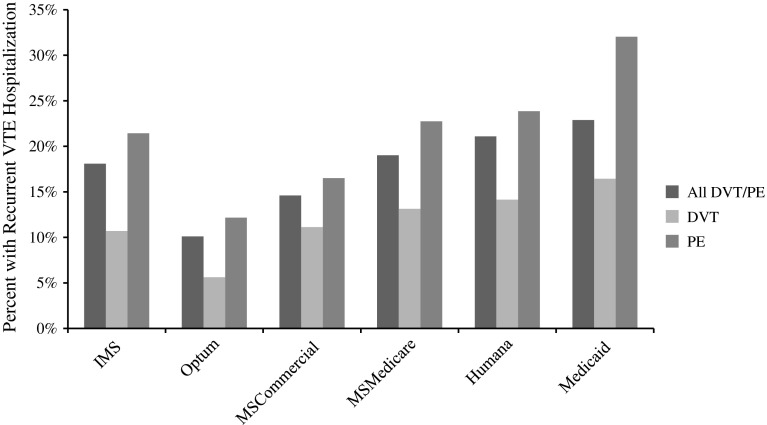
Presence of recurrent VTE hospitalization up to 1 year following index, stratified by VTE type and database. *VTE* venous thromboembolism, *DVT* deep vein thrombosis, *PE* pulmonary embolism IMS, 07/2010–06/2012; Optum, 04/2010–03/2012; MSCommercial, 07/2009–06/2011; MSMedicare, 07/2009–06/2011; Humana, 07/2010–06/2012;Medicaid, 07/2008–06/2010

During the one year follow-up period, recurrent VTE-related hospitalizations (i.e., primary DVT/PE coded inpatient visit after initial index event) occurred in 10 to 23 % of patients across the databases. The highest rates of recurrent VTE-related hospitalizations occurred in the Medicare (19–21 %) and Medicaid (23 %) populations. Approximately 16 % of patients with DVT and 32 % of patients with PE experienced a recurrent VTE-related hospitalization during follow-up in the Medicaid database. In the Medicare databases, 13 and 14 % of DVT and 23 and 24 % of PE patients experienced a recurrent hospitalization in the MSMedicare and Humana populations, respectively. Recurrent event rates among PE patients (12–32 %) were higher than those for DVT patients (6–16 %) across all databases (Fig 1).

### All-cause resource utilization

All-cause hospitalization in the year following the index event was common across payers, with the highest rate among Medicaid patients (67 % overall VTE), and a range of 27–49 % across other databases (Table 3). Rates of hospitalization among PE patients were greater than or equal to those among DVT patients in each study database (range 23–67 % DVT; 30–68 % PE). The mean number of hospitalizations during follow-up among those experiencing a hospitalization was relatively consistent for the DVT (range 1.69–2.34) and PE (range 1.61–2.18) populations across commercial and Medicare databases, and was higher in the Medicaid database (4.68 DVT; 4.7 PE).

**Table 3:** All-cause resource utilization up to 1 year following index among patients with initial VTE event

Measure	IMS			Optum			MSCommercial			MSMedicare			Humana			Medicaid		
	All DVT/PE	DVT	PE	All DVT/PE	DVT	PE	All DVT/PE	DVT	PE	All DVT/PE	DVT	PE	All DVT/PE	DVT	PE	All DVT/PE	DVT	PE
N	1,055	327	728	505	160	345	1,541	548	993	510	198	312	697	198	499	249	146	103
All cause hospitalizations																		
Percentage with ≥ 1 visit during follow-up (%)	38	34	40	27	23	30	32	33	33	43	37	48	49	49	49	67	67	68
Mean number of hospitalizations^a^	1.88	1.91	1.87	1.63	1.69	1.61	1.84	1.95	1.79	1.97	1.91	2.01	2.22	2.34	2.18	4.69	4.68	4.70
30 Days All cause hospitalizations																		
Percentage with ≥1 visit during follow-up (%)	20	18	21	13	12	14	18	18	19	23	17	27	25	27	24	32	30	36
Mean number of visits^a^	1.31	1.32	1.30	1.19	1.24	1.17	1.29	1.29	1.29	1.33	1.23	1.39	1.34	1.36	1.33	1.54	1.50	1.60
All cause ER visits																		
Percentage with ≥1 visit during follow-up (%)	40	36	43	28	27	29	31	29	32	33	32	35	48	50	47	73	72	75
Mean number of visits^a^	2.30	2.10	2.38	1.82	1.88	1.79	2.11	2.01	2.16	2.03	1.94	2.09	2.54	2.67	2.49	6.33	6.23	6.47

*Data source* IMS, 07/2010-06/2012; Optum, 04/2010–03/2012; MSCommercial, 07/2009–06/2011; MSMedicare, 07/2009–06/2011; Humana, 07/2010–06/2012; Medicaid, 07/2008–06/2010

*VTE* venous thromboembolism, *DVT* deep vein thrombosis, *PE* pulmonary embolism, *ER* emergency room

^a^ Among those with an event (i.e., hospitalization or visit)

All-cause hospitalizations within 30 days of the index event occurred in 12–30 % of DVT patients and 14–36 % of PE patients across all databases. All-cause ER visits were also common throughout the study period, occurring in 27–72 % of DVT patients and 29–75 % of PE patients. As with hospitalizations, Medicaid patients had the highest proportion of all-cause ER visits during the year after index VTE diagnosis.

## Discussion

### Summary

Our study used a common analysis platform to assess VTE recurrence rates, all-cause 30 day readmission rates, and all-cause hospitalizations and ER admissions among patients with a diagnosis of DVT or PE using data from commercial, Medicare, and Medicaid payer perspectives. The overall rate of recurrent VTE-related hospitalization was relatively common (i.e., 6–16 % DVT; 12–32 % PE) over the 1-year follow-up period. Rates were typically higher among patients with an index PE event than DVT event, and the highest rates occurred among Medicaid patients. 30-days hospital readmission rates shared a similar likelihood of occurrence, with rates being the highest among patients in the Medicare and Medicaid databases. All-cause hospitalizations and ER visits in the year following index DVT/PE episode were common across all study databases, with approximately one-third of patients experiencing either event during the follow-up period.

### Comparison with other literature

In our analysis, inclusion criteria identified approximately twice as many PE patients as DVT (3,057 vs. 1,590) across all included study databases. In order to ensure that included patients experienced an index event, DVT or PE diagnosis was required to be identified through an inpatient or ER visit. Given that approximately 50 % of DVT patients are treated initially in the outpatient setting [15], this strict inclusion criterion resulted in a VTE population with a different composition than that found in clinical practice, where approximately two-thirds of VTE patients typically have a DVT diagnosis [16].

Although there are few studies that have evaluated these outcomes across multiple payers, our findings are broadly consistent with previously published estimates of VTE recurrence, all-cause and 30 days hospitalization rates, and index event length of stay in a general VTE patient population. A 2005 study conducted by Bullano and colleagues found a rate of recurrence of 13.4 % (12.1 % DVT; 18.5 % PE) over a period of 21 months among commercially insured patients with no prior history of VTE using a more narrow set of VTE diagnosis codes than the present study [9]. This estimate is comparable to the results identified among commercially insured patients in our study over 12 months (e.g., 15 % overall recurrence rate in MSCommercial; 11 % DVT, 17 % PE). Furthermore, Detalziweig 2010, using the Integrated Healthcare Information services database, reported an 11 % recurrent VTE hospitalization rate over a variable follow-up period [17]. Spyropolous et al. [10] evaluated the direct medical costs of subsequent hospital readmissions among patients with VTE from 30 managed care organizations. During a 1 year follow-up period, 19.6 % of patients were rehospitalized with a primary or secondary diagnosis of DVT or PE.

A recent publication by Khorana and colleagues found that commercially insured patients with cancer and VTE experienced an average of 1.38 all-cause hospitalizations during the 12 months after index VTE event [18]. While the patients included in our study were not required to have a diagnosis of cancer, 34–53 % had cancer as a baseline comorbidity, resulting in a similar range among included commercial databases (1.63–1.88). Results for index VTE length of stay and 30 day readmission also are comparable to recently published studies. A 2011 electronic medical record and chart review among patients with a diagnosis of VTE found that insured patients had an average index length of stay of 3.7 days, with approximately 9 % experiencing a hospital readmission within 30 days and 11 % being readmitted to the ER within 30 days [19].

### Implications of our work

The high rate of recurrent VTE-related hospitalizations (10–23 %) and 30 days all-cause hospitalizations (13–32 %) observed in our study represent significant economic issues for both healthcare providers and payers. In addition to the high direct costs associated with recurrent VTE-related hospitalizations (estimated at over $10,000 for DVT and $16,000 for PE) [10], a high rate of rehospitalization has a negative impact on quality of care, which has become an increasingly important metric in the evaluation of healthcare during recent years. In 2008 the Joint Commission, in collaboration with the National Quality Forum, established a VTE prevention and quality measurement initiative. The initiative established six VTE quality measures which aim to reduce the incidence of preventable hospital-acquired DVT and PE, as well as the incidence of VTE readmissions [11]. Quality metrics include items such as adequate venous thromboembolism prophylaxis and accurate anticoagulation instructions at discharge. While not currently tied to reimbursement, these quality measures are collected and published annually by the Joint Commission. The Affordable Care Act has also established Medicare reimbursement penalties for hospitals with excessive re-admission rates within 30 days of hospital discharge. Penalties currently only apply to acute myocardial infarction, heart failure, and pneumonia readmissions; however, additional conditions are expected to be added to the program in upcoming years [12].

Our findings suggest a possible need for both public and private payers to monitor readmission rates among this patient population. A reduction in readmissions could potentially lessen the direct costs associated with VTE for the managed care organization while improving the quality of care provided to the patient. This is particularly important among the Medicaid population, where rates of VTE recurrence over the one year follow-up period were the highest among all analyzed populations (23 % overall VTE population; 16 % DVT; 32 % PE). Given that data on Medicaid-specific VTE recurrence rates are limited in the published scientific literature, our study provides rationale for further investigation among this VTE subpopulation. Additional analyses that aim to determine the sociodemographic and clinical characteristics associated with VTE recurrence among Medicaid patients would help to further understand the results observed in our analysis, and provide useful data for identifying patients at high risk of recurrence.

### Limitations

This study was designed as a descriptive analysis and was not intended to compare outcomes across different cohorts (e.g., techniques such as propensity score matching were not employed). The software tool did not allow analyses of dispersion around the mean values (e.g., using standard deviation or ranges), and continuous patient enrollment during follow-up was not required by the tool, potentially resulting in the underestimation of events and resource use. Furthermore, the primary objective of this study was to analyze the proportion of patients experiencing recurrent VTE events during a 1 year period from multiple payer perspectives. Additional analyses that evaluate the time to recurrent events, predictors of recurrent events/30 days hospitalizations, or the correlation between recurrent events and treatment status among this patient population would provide further insight into the outcomes observed in this study and are warranted.

Due to strict inclusion criteria which identified DVT or PE events based on inpatient or ER claims, patients with initial DVT treatment in an outpatient setting were not included in this study, potentially decreasing the eligible sample size for analysis. Study findings and their implications are therefore limited to DVT and PE patients initially treated in the inpatient/ER setting.

These analyses relied on claims data, which are used primarily for administrative (i.e., billing and operations) purposes and therefore do not reflect all clinical variables that are taken into account by physicians when making treatment decisions. It is also challenging to ensure that subsequent admissions for VTE are new events, as opposed to re-hospitalizations for the index event, when using the limited clinical data available in administrative claims. To address this limitation of the data source, we required that all recurrent VTE events have a primary admitting diagnosis of DVT or PE in an inpatient hospitalization setting. These criteria help to limit the identification of events in which a prior DVT/PE diagnosis is coded during a non-VTE related hospitalization. Furthermore, while the Medicare and commercial databases had broad geographical representation, the Medicaid population analyzed in this study only reflects data collected for a single US state.

## Conclusion

Findings from this analysis demonstrate the high rate of resource utilization and relatively common occurrence of subsequent VTE hospitalizations among patients with an initial VTE event. Results were similar across multiple payer perspectives, a potential observation of interest to clinicians and policymakers charged with improving quality of care among a diverse patient population. Increased use of the analyzer utilized in this analysis and similar software may support enhanced education efforts aimed at improving these outcomes.
